# Effect of Extending the Original Eligibility Criteria for the CROSS Neoadjuvant Chemoradiotherapy on Toxicity and Survival in Esophageal Cancer

**DOI:** 10.1245/s10434-017-5797-3

**Published:** 2017-02-10

**Authors:** E. C. de Heer, J. B. Hulshoff, D. Klerk, J. G. M. Burgerhof, D. J. A. de Groot, J. Th. M. Plukker, G. A. P. Hospers

**Affiliations:** 1 Department of Surgical Oncology, University of Groningen, University Medical Center Groningen, Groningen, The Netherlands; 2 Department of Medical Oncology, University Medical Center Groningen, Groningen, The Netherlands; 3 Department of Epidemiology, University of Groningen, Groningen, The Netherlands

## Abstract

**Background:**

Patients with curable esophageal cancer (EC) who proceed beyond the original Chemoradiotherapy for Oesophageal Cancer Followed by Surgery Study (CROSS) eligibility criteria are also treated with neoadjuvant chemoradiotherapy (nCRT). This study assessed the effect that extending the CROSS eligibility criteria for nCRT has on treatment-related toxicity and overall survival (OS) in EC.

**Methods:**

The study enrolled 161 patients with locally advanced EC (T1N1-3/T2-4aN0-3/M0) treated with the CROSS schedule followed by esophagectomy. Group 1 consisted of 89 patients who met the CROSS criteria, and group 2 consisted of 72 patients who met the extended eligibility criteria, i.e. a tumor length greater than 8 cm (*n* = 24), more than 10% weight loss (*n* = 35), more than 2–4 cm extension in the stomach (*n* = 21), celiac lymph node metastasis (*n* = 13), and/or age over 75 years (*n* = 2). The study assessed the differences in nCRT-associated toxicity [National Cancer Institute’s Common Terminology Criteria for Adverse Events (CTCAE) grade ≥ 3] and 90-day postoperative mortality. Moreover, the prognostic value for OS was assessed with multivariate Cox regression analysis.

**Results:**

No difference was found in nCRT-associated toxicity (*P* = 0.117), postoperative complications (*P* = 0.783), and 90-day mortality (*P* = 0.492). The OS differed significantly (*P* = 0.004), with a median of 37.3 months [95% confidence interval (CI), 10.4–64.2 months] for group 1 and 17.2 months (95% CI 13.8–20.7 months) for group 2. Pathologic N stage (*P* = 0.023), pathologic T stage (*P* = 0.043), and group 2 (*P* = 0.008) were independent prognostic factors for OS.

**Conclusions:**

Extension of the CROSS study eligibility criteria for nCRT did not affect nCRT-associated toxicity, postoperative complications, and postoperative mortality, but was prognostic for OS.

**Electronic supplementary material:**

The online version of this article (doi:10.1245/s10434-017-5797-3) contains supplementary material, which is available to authorized users.

Neoadjuvant chemoradiotherapy (nCRT) according to the Chemoradiotherapy for Oesophageal Cancer Followed by Surgery Study (CROSS) schedule (carboplatin/paclitaxel and 41.4 Gy radiotherapy) followed by a radical surgical resection is the gold standard for locally advanced esophageal cancer (EC) in the Netherlands.^1^ This nCRT scheme increased the 5-year overall survival (OS) by 10–13% while the postoperative complication rate did not increase.^1^,^2^

Patients with a potentially curative resectable EC who do not meet the original CROSS study inclusion criteria are currently also treated with nCRT, i.e. including patients aged over 75 years and those with a tumor length >8 cm, a tumor that extends >2–4 cm into the gastric cardia, and/or >10% body weight loss. Moreover, the original CROSS study excluded patients with celiac lymph node metastases because these nodes were previously classified as distant metastases (M1a) in the American Joint Committee on Cancer (AJCC) TNM 6th edition.^3^ The currently used 7th edition of the AJCC TNM classifies celiac node involvement as regional metastasis (N1–3), and these patients are consequently treated with nCRT.^4^

Besides a small Dutch study, which found that the extended inclusion criteria tumor length >8 cm and age over 75 years did not influence the complication rate, no study has assessed the influence of extension of all CROSS eligibility criteria for nCRT on toxicity and survival.^5^ This study was designed to assess the effect of extended eligibility criteria for treatment with nCRT on the toxicity and mortality (<90 days posttreatment) of EC patients. Furthermore, we assessed the difference in disease-free survival (DFS) and OS between patients that met the original CROSS study inclusion criteria and patients in the extended inclusion group.

## Patients and Methods

### Patients

Data for this retrospective study were obtained from a prospectively maintained database and the study was conducted according to the national guidelines and the rules approved by the local ethics board. All patients with locally advanced EC (TNM7: T1N1-3/T2-4aN0-3/M0) who underwent nCRT according to the CROSS schedule followed by surgery between 2005 and 2015 at the University Medical Center Groningen were eligible for inclusion.

All patients included in the study had a histologically proven adenocarcinoma or squamous cell carcinoma of the esophagus or esophagogastric junction. In addition, the patients had an adequate hematologic, renal, hepatic, and pulmonary function, together with a World Health Organization (WHO) performance status of 2 or lower.

Based on the aforementioned criteria, 177 patients were eligible for inclusion. A total of 16 patients were excluded because of concurrent malignancies (*n* = 3), previous malignancies within 5 years before treatment (*n* = 3), missing blood values (*n* = 7), progressive disease due to distant metastases present on the restaging PET/CT (*n* = 2), or a prolonged interval (>6 months) between nCRT and surgery (*n* = 1). Consequently, 161 patients were included in the study.

## Methods

The patients were divided in two groups. Group 1 consisted of 89 patients who met the original CROSS study inclusion criteria, and group 2 consisted of 72 patients with the extended nCRT criteria. Group 2 included 24 patients with a tumor longer than 8 cm, 35 patients with more than 10% weight loss, 21 patients with more than 2–4 cm tumor extension in the gastric cardia, 13 patients with celiac lymph node metastasis, and 2 patients older than 75 years.

The primary objective was to assess the difference in nCRT-related toxicity (grade ≥ 3) between group 1 and 2. All treatment complications and severity were measured according to the National Cancer Institute’s Common Terminology Criteria for Adverse Events (CTCAE) version 4.0 grading scale.^6^ The secondary outcomes were the difference in postoperative complications, postoperative mortality (30- and 90-day rates), DFS, and OS. DFS was defined as the time between the start of nCRT and the date of tumor recurrence and OS as the time between the start of nCRT and the date of death or last follow-up.

In addition, we compared OS of the extended CROSS group with a reference dCRT group using a multivariate Cox regression analysis containing all confounders (gender, cTN stage, tumor location, tumor length, histology, and age).

### Staging

All patients were staged with endoscopic ultrasonography combined with a fine-needle aspiration biopsy when indicated, computed tomography (CT) of the chest and abdomen, and 18-F-fluorodeoxyglucose (FDG) positron emission tomography (PET) or integrated FDG-PET/CT. When indicated, additional imaging was performed. Patients were staged according to the 7th edition of the tumor-node-metastasis (TNM) classification.^4^

### Treatment

All patients received nCRT according to the CROSS schedule, consisting of five weekly intravenous administrations of carboplatin [area under the curve (AUC) 2 mg/ml/min] and paclitaxel (50 mg/m^2^), as well as concurrent external beam radiotherapy (41.4 Gy/23 fractions) 5 days per week.^1^,^2^ After nCRT, either a radical transthoracic or minimally invasive esophagectomy was performed, with en bloc dissection of mediastinal and abdominal lymph nodes. Definitive chemoradiotherapy (dCRT) consisted of either carboplatin/paclitaxel (AUC 2 and 50 mg/m^2^) or cisplatin and fluorouracil (Cis-5FU, 75 mg/m^2^ and 1 g/m^2^) combined with radiotherapy (40–60 Gy in 30 fractions).

### Pathology

Resected specimens were pathologically assessed according to a standard protocol on histologic subtype, radicality of the resection margins (proximal, distal, and circumferential), pathologic T (ypT) stage, pathologic lymph node (ypN) stage, tumor location, perineural growth, and lymphangio-invasion.

### Follow-up Evaluation

According to the standard protocol, patients were seen every 3 months during the first year, every 4 and 6 months during the second and third year, and subsequently once every succeeding year until 10 years after treatment. During the follow-up, tumor recurrence and/or cause of death was accurately described. Tumor recurrence was proven either pathologically or radiologically.

### Statistical Analysis

Differences in patient characteristics and complications were assessed using the Chi square test or the likelihood ratio test for categorical variables and the Mann–Whitney *U* test for non-normally distributed variables.

Kaplan–Meier curves were used to display the DFS and OS. Univariate Cox regression analysis was performed on all possible prognostic factors for both DFS and OS. All factors with a *P* value lower than 0.10 in the univariate Cox regression analysis were included in the multivariate Cox regression analysis. A *P* value lower than 0.05 was considered statistically significant. All statistical analyses were performed with IBM SPSS Statistics for Windows, version 22.0 (IBM Corp., Armonk, NY, USA).

## Results

### Patients’ Characteristics

The characteristics of the patients are summarized in Table 1. The group 2 patients (*n* = 72) were more likely to have a tumor involving the gastroesophageal (GE) junction (*P* = 0.005), a higher clinical T stage (cT; *P* = 0.000), and a higher clinical N stage (*P* = 0.024) than the group 1 patients (*n* = 89). In addition, significantly more patients in group II died (*P* = 0.004) and the follow-up period was significantly shorter for group 2, with a median follow-up of 16.2 months [interquartile range (IQR) 9.2–40.3 months] compared with 23.2 months (IQR 11.8–52.9 months) for group 1 (*P* = 0.037).

**Table 1:** Patient and tumor characteristics of group 1 (CROSS inclusion criteria) and group 2 (not eligible for CROSS)

	Group 1 (*n* = 89) *n* (%)	Group 2 (*n* = 72) *n* (%)	*P* value
Male	71 (79.8)	57 (79.2)	0.924^a^
Age (years), median (IQR)	63 (58–67)	64 (57–69)	0.299^b^
WHO/ECOG performance status			0.843^a^
0–1	85 (95.5)	64 (88.9)	
2	0 (0.0)	0 (0.0)	
Missing	4 (4.5)	8 (11.1)	
Comorbidities total	44 (49.4)	38 (52.8)	0.673^a^
Cardiovascular	34 (38.2)	28 (38.9)	0.798^a^
Pulmonary	3 (3.4)	1 (1.4)	
Cardiovascular and pulmonary	5 (5.6)	6 (8.3)	
Other	2 (2.2)	3 (4.2)	
No comorbidities	45 (50.6)	34 (47.2)	
Histology			0.095^a^
Adenocarcinoma	79 (88.8)	57 (79.2)	
Squamous cell carcinoma	10 (11.2)	15 (20.8)	
Tumor location			
Middle esophagus	7 (7.9)	5 (6.9)	0.005^a^
Distal esophagus	76 (85.4)	49 (68.1)	
GEJ	6 (6.7)	18 (25.0)	
Tumor length (cm), median (IQR)	5.0 (3.0–6.0)	6.5 (5.0–9.0)	0.000^b^
cT stage			0.000^a^
T1	0 (0.0)	2 (2.8)	
T2	25 (28.1)	5 (6.9)	
T3	63 (70.8)	56 (77.8)	
T4a	1 (1.1)	9 (12.5)	
cN stage			0.024^a^
N0	22 (24.7)	7 (9.7)	
N1	38 (42.7)	30 (41.7)	
N2	27 (30.3)	29 (40.3)	
N3	2 (2.2)	6 (8.3)	
ypT stage			0.525^a^
CR	15 (16.9)	13 (18.1)	
T0	4 (4.5)	2 (2.8)	
T1	17 (19.1)	8 (11.1)	
T2	11 (12.4)	9 (12.5)	
T3	42 (47.2)	40 (55.6)	
ypN stage			0.706^a^
N0	57 (64.0)	44 (61.1)	
N1	18 (20.2)	16 (22.2)	
N2	11 (12.4)	7 (9.7)	
N3	3 (3.4)	5 (6.9)	
Perineural growth	15 (16.9)	18 (25.0)	0.204^a^
Lymphangio-invasion	14 (15.7)	19 (26.4)	0.097^a^
LN ratio (>0.2 LN+)	12 (13.5)	11 (15.3)	0.747^a^
Follow-up (months), median (IQR)	23.2 (11.8–52.9)	16.2 (9.2–40.3)	0.037^b^

*IQR* interquartile range, *WHO* World Health Organization, *ECOG* Eastern Cooperative Oncology Group, *GEJ* gastroesophageal junction, *cT* clinical T stage, *cN* clinical N stage, *ypT* pathologic T stage, *ypN* pathologic lymph node stage, *LN* lymph node

^a^Likelihood ratio

^b^Mann–Whitney *U* test

In group 1 and 2 respectively 79.8 and 80.6% of the patients were able to complete the entire nCRT regimen (Table 2). Of the patients in group 2, 12 (16.7%) fulfilled two extended criteria, 4 (5.6%) fulfilled three criteria, and 1 (1.4%) fulfilled four criteria. The presence of two or more extended eligibility criteria within a patient (*n* = 17) versus only one extended criterion (*n* = 55) did not influence the OS (*P* = 0.642) or DFS (*P* = 0.198).

**Table 2:** Treatment toxicity and complications

	Group 1 (*n* = 89) *n* (%)	Group 2 (*n* = 72) *n* (%)	*P* value
Completed nCRT	71 (79.8)	58 (80.6)	0.902^a^
Hematologic toxicity			0.068^a^
Thrombocytopenia–overall			
Not applicable	26 (29.2)	28 (38.9)	
Grade 1	54 (60.7)	43 (59.7)	
Grade 2	8 (9.0)	1 (1.4)	
Grade 3	1 (1.1)	0 (0.0)	
Leukopenia–overall			0.338^a^
Not applicable	15 (16.9)	20 (27.8)	
Grade 1	34 (38.2)	21 (29.2)	
Grade 2	26 (29.2)	19 (26.4)	
Grade 3	13 (14.6)	12 (16.7)	
Grade 4	1 (1.1)	0 (0.0)	
Blood transfusion			0.417^a^
0	87 (97.8)	67 (93.1)	
1	0 (0.0)	1 (1.4)	
2	1 (1.1)	1 (1.4)	
3	1 (1.1)	2 (2.8)	
4	0 (0.0)	1 (1.4)	
Other nCRT complications (grade ≥ 3)			
Anemia	0 (0.0)	0 (0.0)	NA^a^
Bleeding	0 (0.0)	1 (1.4)	0.203^a^
Nausea	3 (3.4)	4 (5.6)	0.501^a^
Fatigue	1 (1.1)	1 (1.4)	0.880^a^
Neurotoxic	0 (0.0)	2 (2.8)	0.071^a^
Diarrhea	0 (0.0)	1 (1.4)	0.203^a^
Esophagitis	2 (2.2)	5 (6.9)	0.144^a^
Grade ≥ 3 or blood transfusion	22 (24.7)	26 (36.1)	0.117^a^
Postoperative complications			
Pulmonary (all grades)^b^	49 (55.1)	38 (52.8)	0.773^a^
Pneumonia	41 (46.1)	28 (38.9)	0.360^a^
Respiratory insufficiency	19 (21.3)	13 (18.1)	0.602^a^
Pulmonary embolism	2 (2.2)	0 (0.0)	0.122^a^
Cardiac (all grades)^c^	26 (29.2)	22 (30.6)	0.835^a^
Arrhythmia	25 (28.1)	22 (30.6)	0.732^a^
Myocardial infarction	1 (1.4)	0 (0.0)	0.273^a^
Sepsis	8 (9.0)	6 (8.3)	0.883^a^
Postoperative bleeding	2 (2.2)	1 (1.4)	0.678^a^
Chylothorax	11 (12.4)	3 (4.2)	0.057^a^
Cardiac arrest	2 (2.2)	3 (4.2)	0.486^a^
Esophageal anastomotic leak	8 (9.0)	12 (16.7)	0.143^a^
Renal failure	2 (2.2)	4 (5.6)	0.276^a^
IIeus	6 (6.7)	2 (2.8)	0.237^a^
All patients with complications (all grades)	60 (67.4)	50 (69.4)	0.783^a^
Postoperative mortality			
30-day mortality	2 (2.2)	3 (4.2)	0.486^a^
90-day mortality	6 (6.7)	7 (9.7)	0.492^a^

*nCRT* neoadjuvant chemoradiotherapy, *NA* not applicable

^a^Likelihood ratio

^b^Pneumonia, atelectasis, respiratory insufficiency, acute respiratory distress syndrome, pleural effusion, pneumothorax and/or pulmonary embolism

^c^Arrhythmia and/or myocardial infarction

### Toxicity and Postoperative Survival

Table 2 displays the distribution of nCRT toxicity, postoperative complications, and postoperative mortality (30- and 90-day rates) between the two groups. A total of 48 patients (29.8%) experienced severe toxicity (grade ≥ 3) or received a blood transfusion. The total toxicity rates did not differ between the two groups (*P* = 0.117), nor did the number of postoperative complications (data shown in Table 2).

Although more patients in group 2 (*n* = 7, 9.7%) died within 90 days after surgery than in group 1 (*n* = 6, 6.7%), this difference was not significant (*P* = 0.492). In addition, the 30-day postoperative mortality did not differ between the two groups (*P* = 0.486), with a 30-day mortality rate of 2.2% (*n* = 2) in group 1 and 4.2% (*n* = 3) in group 2.

### Overall Survival

**Fig. 1:**
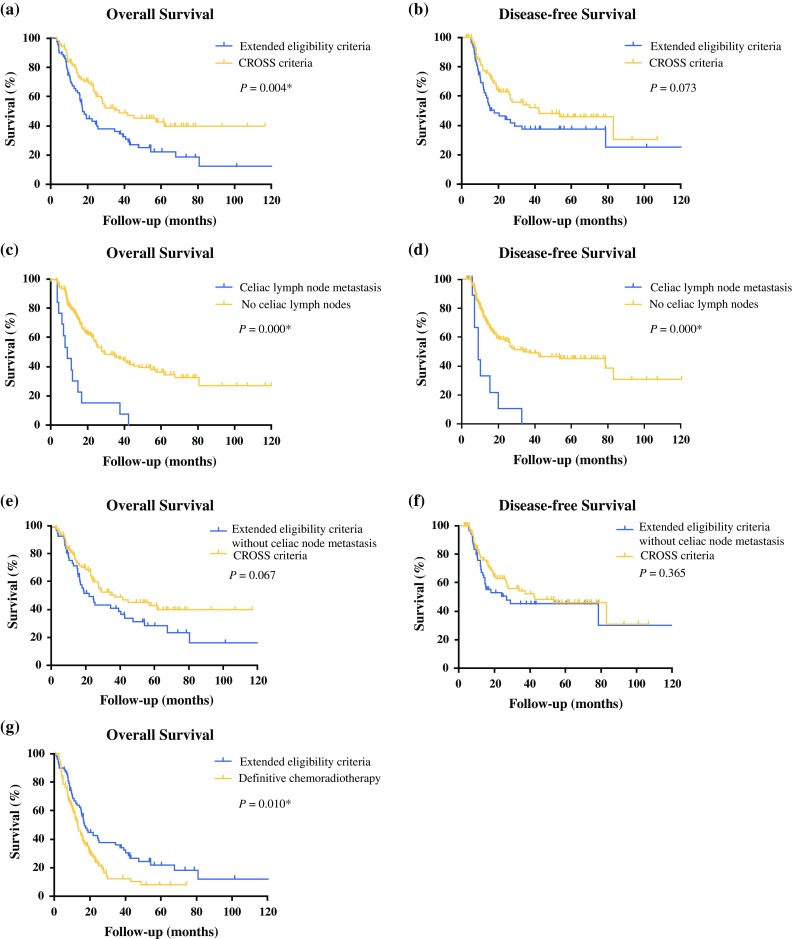
The overall and disease-free survival in patients that met the original CROSS criteria or the extended CROSS eligibility criteria (**a, b**), in patients with or without celiac lymph node metastases (**c, d**), and in patients that met the original CROSS criteria or the extended CROSS eligibility criteria without celiac lymph node metastases (**e, f**). And the overall survival in patients that met the extended CROSS eligibility criteria or patients from a definitive chemoradiotherapy reference group (**g**)

Figure 1 displays the Kaplan–Meier curves with the OS and DFS for both group 1 and 2. The OS differed significantly between the two groups (*P* = 0.004: Fig. 1a), with a median of 37.3 months (95% confidence interval [CI] 10.4–64.2 months) in group 1 and 17.2 months (95% CI 13.8–20.7 months) in group 2. Table 3 displays the extended CROSS criteria and the factors with a *P* value lower than 0.10 in the univariate analysis. Independent prognostic factors for OS in the multivariate Cox regression analysis were ypN (*P* = 0.023), ypT (*P* = 0.043), and group 2 (*P* = 0.008). In a multivariate Cox regression analysis that assessed each eligibility criterion separately, only celiac lymph node involvement [hazard ratio (HR) 3.583; 95% CI 1.884–6.814; *P* = 0.000] was an independent prognostic factor for OS.

**Table 3:** Prognostic factors on uni- and multivariate Cox regression analysis for overall survival

	HR (95% CI)	*P* value
Univariate analysis		
Group 2	1.802 (1.200–2.707)	0.005
Celiac lymph node metastasis	3.969 (2.188–7.198)	0.000
Cardia growth 2–4 cm	1.329 (0.721–2.452)	0.362
Length >8 cm	1.217 (0.699–2.118)	0.488
Weight loss >10%	1.407 (0.892–2.217)	0.142
Squamous cell carcinoma	0.543 (0.295–1.000)	0.050
ypT0	1.000	0.008
ypT1	0.589 (0.254–1.367)	
ypT2	1.945 (0.974–3.884)	
ypT3	1.778 (1.019–3.100)	
ypN0	1.000	0.000
ypN1	1.518 (0.914–2.522)	
ypN2	2.144 (1.158–3.968)	
ypN3	5.024 (2.215–11.398)	
R1 resection	3.266 (1.543–6.912)	0.002
LN ratio (>0.2 LN +)	2.29 (1.437–4.105)	0.001
Perineural growth	2.076 (1.314–3.279)	0.002
Lymphangio-invasion	1.829 (1.125–2.874)	0.015
Multivariate analysis^a^		
ypT0	1.000	0.043
ypT1	0.540 (0.224–1.301)	
ypT2	1.798 (0.854–3.789)	
ypT3	1.294 (0.704–2.378)	
Group 2	1.762 (1.157–2.685)	0.008
ypN0	1.000	0.023
ypN1	1.349 (0.805–2.263)	
ypN2	1.896 (0.989–3.635)	
ypN3	3.415 (1.446–8.064)	

*HR* hazard ratio, *CI* confidence interval, *ypT* pathologic T stage, *ypN* pathologic lymph node stage, *LN* lymph node

^a^Variables with *P* < 0.10 in the univariate analysis were included in the multivariate analysis

### Disease-Free Survival

The difference in DFS between group 1 and 2 approached significance (*P* = 0.073; Fig. 1b), with a median of 42.5 months (95% CI 15.7–69.4 months) in group 1 and 18.2 months (95% CI 7.4–28.9 months) in group 2. Table 4 displays the extended CROSS criteria and the factors with a *P* value lower than 0.10 in the univariate analyses, as well as the independent prognostic factors in the multivariate Cox regression analysis for DFS. Gender (*P* = 0.024), LN ratio (*P* = 0.001), squamous cell carcinoma (*P* = 0.031), and group 2 (*P* = 0.027) were independent prognostic factors for DFS. A closer look at specific subgroups of group 2 with multivariate Cox regression analysis showed that only celiac lymph node metastasis was an independent prognostic factor for DFS (HR 3.741; CI 1.822–7.680; *P* = 0.000).

**Table 4:** Prognostic factors on uni- and multivariate Cox regression analysis for disease-free survival

	HR (95% CI)	*P* value
Univariate analysis		
Group 2	1.509 (0.959–2.375)	0.075
Celiac lymph node metastasis	3.898 (1.923–7.904)	0.000
Cardia growth 2–4 cm	1.454 (0.742–2.849)	0.275
Length >8 cm	1.103 (0.580–2.097)	0.764
Weight loss >10%	1.229 (0.720–2.096)	0.450
Female	0.484 (0.255–0.920)	0.027
Squamous cell carcinoma	0.366 (0.167–0.802)	0.012
cT1 and T2	1.000	0.084
cT3	1.961 (1.003–3.833)	
cT4a	2.894 (0.984–8.510)	
ypT0	1.000	0.023
ypT1	1.535 (0.636–3.706)	
ypT2	3.056 (1.298–7.194)	
ypT3	2.632 (1.275–5.435)	
ypN0	1.000	0.001
ypN1	1.470 (0.812–2.659)	
ypN2	3.060 (1.618–5.785)	
ypN3	4.374 (1.682–11.375)	
R1 resection	4.389 (2.043–9.431)	0.000
LN ratio (>0.2 LN +)	3.106 (1.758–5.489)	0.000
Perineural growth	1.694 (0.993–2.890)	0.053
Lymphangio-invasion	1.940 (1.131–3.327)	0.016
Multivariate analysis^a^		
Female	0.474 (0.248–0.907)	0.024
Squamous cell carcinoma	0.413 (0.185–0.923)	0.031
Group 2	1.685 (1.061–2.676)	0.027
LN ratio (>0.2 LN +)	2.712 (1.524–4.826)	0.001

*HR* hazard ratio, *CI* confidence interval, *cT* clinical T stage, *ypT* pathologic T stage, *ypN* pathologic lymph node stage, *LN* lymph node

PUB1: OK not to spell PET/CT here? Spelling here would be cumbersome

^a^Variables with *P* < 0.10 in the univariate analysis were included in the multivariate analysis

### Comparison of Survival Between the Extended CROSS and dCRT Reference Group

Supplementary Table 1 depicts the characteristics of the dCRT and extended CROSS group. The dCRT group (*n* = 80) and the extended CROSS group (*n* = 72) differed in cT stage (*P* = 0.001), cN stage (*P* = 0.000), squamous cell carcinoma (*P* = 0.006), tumor location (*P* = 0.001), age (*P* = 0.021), and WHO performance status (*P* = 0.007). The patients in the extended CROSS group showed an increased OS (*P* = 0.010; Fig. 1g) with the log-rank test but not in the Cox-regression model (Supplementary Table 2) that contained possible confounders. The number of complications grade ≥ 3 did not differ between the two groups (*P* = 0.115).

## Discussion

Several randomized studies, including the CROSS study, have shown that nCRT increases both OS and DFS for EC patients with locoregional disease compared with surgery alone.^1^,^7^ Moreover, pathologic complete response rates of approximately 30% are commonly observed after nCRT.^1^ Extending the original criteria for CROSS nCRT is a logical step to improvement of survival in locally advanced EC.

In this study, we assessed the impact of extended eligibility criteria for nCRT on toxicities, OS, and DFS in these patients. No difference was found in the toxicity rates between the patients in group 1 (original CROSS criteria) and group 2 (extended CROSS criteria). However, the OS and DFS in group 2 were significantly lower in the multivariate Cox regression analysis.

Schrauwen et al.^5^ (*n* = 116) found that the extended inclusion criteria based on tumor length greater than 8 cm (*n* = 7) and age over 75 years (*n* = 9) had no influence on the complication rates but were prognostic for OS with the log-rank test. However, interpreting these results is difficult due to the low number of patients, the absence of multivariate analysis, and the absence of celiac lymph node metastases in the analysis.^5^

The overall rate of toxicity (grade ≥ 3) or blood transfusion was not significantly higher in group 2 (24.7%) than in group 1 (36.1%) (*P* = 0.117). The incidences of severe leukopenia (grade ≥ 3) in group 1 (15.7%) and group 2 (16.7%) were somewhat higher than the 6% in the original CROSS trial but within the range of 3–24% in the literature.^1^,^8^,^9^ Furthermore, the observed rates of thrombocytopenia grade 3 or higher of 1.1% in group 1 and 0% in group 2 correspond well with the 1% rate of thrombocytopenia in the CROSS trial. The 30-day mortality rates in group 1 (2.2%) and group 2 (4.2%) are also comparable with the mortality rate of 2% in the original CROSS study.^1^ Thus, the CROSS nCRT schedule in group 2 is not associated with significantly higher hematologic or non-hematologic toxicity and can be safely applied in the extended patient category.

The 5-year OS of 47% (median 48.6 months) found in the Dutch randomized CROSS trial is comparable with the 43% (median 37.3 months) in our group 1.^1^,^2^ Conversely, the extended criteria group 2 had a remarkably lower 5-year OS of 23% (median, 17.2 months). The median survival after noninvasive dCRT, an alternative for patients with considerable comorbidity, is 16–21 months, raising the question whether dCRT is worth considering for the extended patient category.^10^–^13^ Nevertheless, direct comparison of survival rates in the dCRT and extended CROSS group is not possible because dCRT studies also included irresectable tumors and inoperable patients.

In the included dCRT reference group, we found a significantly lower OS (*P* = 0.010) with the univariate log-rank test. However, this test does not correct for baseline differences (gender, cTN stage, tumor localization, tumor length, histology, and age) between the extended nCRT group and the dCRT group. Hence, a multivariate Cox regression analysis containing these confounding variables was performed in which the OS did not differ (*P* = 0.445) between the extended CROSS group and the dCRT group. This suggests that the difference in survival curves might be caused by baseline differences between the groups rather than superiority of nCRT followed by surgery over dCRT.

Several studies found a comparable outcome in patients with celiac and regional lymph node metastasis. Celiac lymph node metastases are therefore currently classified as regional lymph nodes (N +), whereas previous classification systems regarded them as distant (M1a).^14^–^16^ In the current study, the presence of tumor-positive celiac lymph nodes (*n* = 13) was the only extended eligibility criterion with an independent prognostic value. We compared the survival of patients with celiac lymph node metastases in the extended CROSS group (*n* = 13) with M1a patients in the dCRT group (the latter involving both irresectable higher mediastinal and celiac nodes; *n* = 15) and found no difference in survival (*P* = 0.336). However, the groups were too small for a solid conclusion. Davies et al.^10^ found that celiac lymph node metastasis (determined by endoscopic ultrasound) was not prognostic for OS after dCRT, which was confirmed by Gwynne et al.^13^ However, further research seems necessary to elucidate the value of dCRT for patients with celiac lymph node metastasis, probably in a randomized controlled trial or a large retrospective study.

The potential limitations of our study include the small sample size, especially the subgroup of patients with celiac lymph node metastases (*n* = 13). Moreover, two of these patients died within 90 days after surgery, which may have influenced the OS. Another potential weakness is that we included only patients who received surgery, whereas approximately 8% experience interval metastases between nCRT and surgery.^17^

In conclusion, extension of the original CROSS inclusion criteria for nCRT followed by surgery in EC did not influence the toxicity rate, indicating safe application of the CROSS nCRT regimen in the extended patient category. However, the OS in the extended CROSS group was significantly lower than in the standard CROSS group and did not differ significantly from the OS in the dCRT reference group in the multivariate Cox regression analysis. This implies that the additional value of nCRT followed by surgery compared with dCRT in the extended CROSS group might be limited. The findings of this study support further research regarding the strategy to extend the original CROSS criteria for nCRT in patients with locally advanced EC, and should focus more on patients with celiac node metastases.

## Electronic supplementary material

Below is the link to the electronic supplementary material.

Supplementary material 1 (DOCX 19 kb)
