# Supplementary material for: Effect of Extending the Original Eligibility Criteria for the CROSS Neoadjuvant Chemoradiotherapy on Toxicity and Survival in Esophageal Cancer
Source: Ann Surg Oncol. 2017 Feb 10;24(7):1811–20. doi: 10.1245/s10434-017-5797-3 (PMC5486922; doi:10.1245/s10434-017-5797-3)
Supplement: Supplementary file 1 — Supplementary material 1 (DOCX 19 kb) [file 10434_2017_5797_MOESM1_ESM.docx]

**Supplementary table 1. Patient and tumor characteristics of the extended CROSS group and the definitive chemoradiotherapy group**

|  | Group II  (n=72), n (%) | dCRT group (n=80), n (%) | *P*-value |
| --- | --- | --- | --- |
| Male | 57 (79.2%) | 61 (76.3%) | P=0.666 ^a^ |
| Age in years; median (IQR) | 64 (57 - 69) | 66 (61 - 73) | *P=0.021* ^b^ |
| WHO/ECOG Performance Status  0-1  2  Missing | 64 (88.9%)  0 (0.0%)  8 (11.1%) | 72 (90.0%)  6 (7.5%)  2 (2.5%) | *P=0.007* ^a^ |
| Comorbidities total | 38 (52.8%) | 42 (52.5%) | P=0.973 ^a^ |
| Cardiovascular  Pulmonary  Cardiovascular & Pulmonary  Other  No comorbidities | 28 (38.9%) 1 (1.4%) 6 (8.3%) 3 (4.2%) 34 (47.2%) | 22 (27.5%) 5 (6.3%) 4 (5.0%) 11 (13.8%) 38 (47.5%) | *P=*0.069 ^a^ |
| Histology:  Adenocarcinoma  Squamous cell carcinoma | 57 (79.2%) 15 (20.8%) | 47 (58.8%) 33 (41.3%) | *P=0.006* ^a^ |
| Tumor location:   Middle esophagus  Distal esophagus  GEJ | 5 (6.9%) 49 (68.1%) 18 (25.0%) | 22 (27.5%) 49 (61.3%) 9 (11.3%) | *P*=*0.001* ^a^ |
| Tumor length (cm); median (IQR) | 6.5 (5.0 - 9.0) | 6.0 (4.0 - 8.0) | P=0.093 ^b^ |
| cT-stage   T1  T2  T3  T4a  missing cN-stage  N0  N1  N2  N3 | 2 (2.8%) 5 (6.9%) 56 (77.8%) 9 (12.5%) 0 (0.0%)  7 (9.7%) 30 (41.7%) 29 (40.3%) 6 (8.3%) | 1 (1.3%) 4 (5.0%) 39 (48.8%) 32 (40.0%) 4 (5.0%)  14 (17.5%) 61 (76.3%) 4 (5.0%) 1 (1.3%) | *P=0.001* ^a^    *P=0.000* ^a^ |
| ≥ Grade 3 or blood transfusion | 26 (36.1%) | 39 (48.8%) | *P*=0.115 ^a^ |
| Follow-up in months; median (IQR) | 16.2 (9.2 - 40.3) | 13.8 (8.0 – 22.1) | *P*=*0.060* ^b^ |

Abbreviations: dCRT = definitive chemoradiotherapy, IQR = interquartile range, GEJ = gastroesophageal junction, cT = clinical T-stage, cN = clinical N-stage. ^a^ = Likelihood Ratio, ^b^ = Mann–Whitney U test.

**Supplementary table 2. Multivariate Cox regression analysis for overall survival in the extended CROSS and definitive chemoradiotherapy group**

| **Overall survival** | | |
| --- | --- | --- |
|  | HR (95% CI) | *P*-value |
| Age >75 | 0.669 (0.321-1.393) | 0.283 |
| Female | 0.497 (0.281-0.881) | *0.017* |
| Tumor length | 0.967 (0.892-1.048) | 0.410 |
| Squamous cell carcinoma | 0.551 (0.307-0.988) | *0.046* |
| cT1 & T2 cT3 cT4a | 1.000  0.584 (0.257-1.330) 1.019 (0.394-2.636) | 0.062 |
| cN0 cN1 cN2 & cN3 | 1.000 0.581 (0.314-1.074) 0.726 (0.346-1.522) | 0.197 |
| Mid Distal  GEJ | 1.000  0.460 (0.238-0.888) 0.337 (0.145-0.783) | *0.031* |
| nCRT dCRT | 1.00 1.233 (0.720-2.113) | 0.445 |

All possible cofounding variables for OS were included in the multivariate models. Abbreviations: HR= hazard ratio, CI = confidence interval, GEJ = gastroesophageal junction, cT = clinical T-stage, cN = clinical N-stage, nCRT = neoadjuvant chemoradiotherapy, and dCRT = definitive chemoradiotherapy.
